# Evaluating User Engagement With a Real-Time, Text-Based Digital Mental Health Support App: Cross-Sectional, Retrospective Study

**DOI:** 10.2196/66301

**Published:** 2025-04-14

**Authors:** Edward Coffield, Khadeja Kausar

**Affiliations:** 1 Department of Population Health Hofstra University Hempstead, NY United States; 2 Business Intelligence and Value Based Management Analytics Medical Affairs Maimonides Medical Center Brooklyn, NY United States

**Keywords:** mental health support, text, app, utilization, mobile, on demand, scheduled, mHealth, mobile health, app, student, university, college, mental health, employee, job, work, occupational health, counselor, counseling, usage, engagement, self-reported

## Abstract

**Background:**

Approximately 20% of US adults identify as having a mental illness. Structural and other barriers prevent many people from receiving mental health services. Digital mental health apps that provide 24-hour, real-time access to human support may improve access to mental health services. However, information is needed regarding how and why people engage with licensed counselors through a digital, real-time, text-based mental health support app in nonexperimental settings.

**Objective:**

This study aimed to evaluate how people engage with Counslr, a 24-hour, digital, mental health support app where users communicate in real time with human counselors through text messaging. Specifically, access patterns (eg, day of the week and time of session) and reasons for accessing the platform were examined. Furthermore, whether differences existed between session types (on-demand or scheduled) and membership types (education or noneducation) in regard to access patterns and why people accessed the platform were evaluated.

**Methods:**

The study population (users) consisted of students whose schools, universities, or colleges partnered with Counslr and employees whose organizations also partnered with Counslr. Users participated in text-based mental health support sessions. In these sessions, users engaged with licensed counselors through digital, text-based messaging in real time. Users could initiate an on-demand session or schedule a session 24 hours a day. User engagement patterns were evaluated through session length, session day, session time, and self-reported reasons for initiating the session. The data were stratified by membership type (education [students] or noneducation [employees]) and session type (on-demand or scheduled) to evaluate whether differences existed in usage patterns and self-reported reasons for initiating sessions by membership and session types.

**Results:**

Most students (178/283, 62.9%) and employees (28/44, 63.6%) accessed Counslr through on-demand sessions. The average and median session times were 40 (SD 15.3) and 45 minutes. On-demand sessions (37.9 minutes) were shorter (*P*=.001) than scheduled sessions (43.5 minutes). Most users (262/327, 80.1%) accessed Counslr between 7 PM and 5 AM. The hours that users accessed Counslr did not statistically differ by membership type (*P*=.19) or session type (*P*=.10). Primary self-reported reasons for accessing Counslr were relationship reasons, depression, and anxiety; however, users initiated sessions for a variety of reasons. Statistically significant differences existed between membership and session types (*P*<.05) for some of the reasons why people initiated sessions.

**Conclusions:**

The novel findings of this study illustrate that real-time, digital mental health support apps, which offer people the opportunity to engage with licensed counselors outside of standard office hours for a variety of mental health conditions, may help address structural barriers to accessing mental health support services. Additional research is needed to evaluate the effectiveness of human-based apps such as Counslr and whether such apps can also address disparities in access to mental health support services among different demographic groups.

## Introduction

### Overview

Approximately 1 out of every 5 adults (22.8%) in the United States identified as having a mental health illness in 2021 [1]. The gap between people who identify as having a mental illness and those who receive care or have all of their mental health care needs met is substantial. Among people who identify as having any mental illness, 47.2% received mental health treatments in 2021 and 27.6% perceived themselves as having an unmet mental health care need [1]. Access to mental health care services is not consistent among groups. For example, adults who identify as non-Hispanic Black or Hispanic are less likely than adults who identify as White to receive mental health care treatments [2].

Possible factors for having an unmet mental health care need include cost, stigma, minimization of symptoms, low perceived treatment effect, and structural barriers [3,4]; these factors differ across demographic groups [3,5]. For instance, the odds of costs being a factor in having an unmet mental health care need are higher among people who identify as female relative to people who identify as male, while the odds of people stating either stigma or structural barriers as a reason for their unmet mental health care needs are higher among people who identify as Black or Hispanic relative to people who identify as White [3].

Digital-based mental health apps have the potential to help people overcome barriers to obtaining care for mental illness [6,7] and reduce unmet mental health care needs among marginalized groups [8]. For example, the ability to consult with mental health professionals at all hours of the day, from any location, and in a digital environment that may foster privacy and anonymity, could address structural barriers and stigma [9]. Furthermore, Friis-Healy et al [10] state that digital mental health tools may help expand access to people most affected by COVID-19 and systematic racism by lowering care costs; “...decreasing transportation challenges; and providing mental health care in a private, destigmatizing manner.” ([10], pages 2-3).

While digital mental health apps have been found to be effective and cost-effective [11], evidence regarding the effectiveness of digital mental health apps is inconsistent across digital mental health app types (eg, guidance within an app likely matters [7,12]). Furthermore, challenges, such as user engagement [6,9,13], remain. Baumel et al [13]—in an evaluation of 93 digital mental health care apps—found that only 4% of users opened the evaluated apps daily. Accordingly, understanding how people engage with digital mental health care apps is needed to facilitate design elements that may encourage engagement. As user engagement with mental health apps may differ between practical settings and clinical trials [6,13], a need exists to understand user engagement in a practical or actual setting. Engagement [13] and effectiveness [7] may also differ by app type, indicating an additional need to understand how people engage with different types of digital mental health apps in a practical setting.

Despite the need for nonexperimental evaluations of digital mental health apps, studies [13,14] regarding how people engage with digital mental health apps in practical settings [15] remain scarce. Studies have evaluated how users interact with (eg, downloading, using, and engaging) nonexperimental mental health apps in various forms [13-17]. For instance, Baumel et al [13] calculated usage metrics (eg, number of user sessions and minutes of daily use) for mobile apps— including 2 apps with peer support as a primary technique (and 7 apps with peer support as a cotechnique, for a total of 9), where the support could be “through chat or messaging services (synchronous and asynchronous...)” ([13], Appendix 2)—with at least 10,000 installs; Booth et al [14] calculated interactions by the hour and other engagement metrics for a chatbot; and Raue et al [17] reported engagement metrics (eg, weekly text message word count and weekly text messages) among users of a text-based app where users send text messages to therapists “24/7” ([17], page 3) who then review messages during “standard working hours” and respond at least “once a day, 5 days a week” ([17], page 3). However, usage patterns may differ between app designs (eg, human-based synchronous platforms, human-based asynchronous platforms, hybrid human-based designs, artificial intelligence [AI]–based platforms, and AI and human–based platforms), creating a need for further study. Notably, information is needed in terms of how (eg, day of the week, hours of day, and length of sessions) and why users engage with human-based, nonscripted apps, where users engage with humans through text messaging in real time. The addition of this information will provide policy makers and app designers with further data to consider when designing mental health apps.

In this study, we evaluate how users engage with Counslr, a digital, text-based mental health support app where users interact with licensed counselors in real time on their mobile devices [18]. This nonexperimental study is novel in that it evaluates how users engage with a digital mental health app that provides real-time, text-based mental health support. In addition, we explore why people access the app and whether engagement patterns statistically differ among people who access Counslr through their employment relative to people who access Counslr through their schools, universities, or colleges. Evaluating how users engage with and use Counslr in this retrospective study will contribute to the literature by providing an examination of how people interact with a counselor-based, digital mental health support app in a practical setting. In addition, this study will provide insights into whether people access a real-time, human-interfacing mobile mental health support app outside of standard business hours and how usage patterns may differ between instant, on-demand access and scheduled appointment access.

### Counslr

Counslr was founded in 2019 and delivers mental health support services to users located in the United States through its mobile, text-based support platform. Through the Counslr platform, licensed counselors are available for confidential, real-time, text-based assistance 24 hours a day, 365 days a year. Counslr now uses an algorithm to match users with counselors based upon a user’s preferences and needs. People may also schedule future sessions with a requested counselor. In addition to the text-based support sessions, Counslr provides self-guided resources within the platform. Access to Counslr is provided through school-, employment-, and community-based organizations that purchase a subscription to Counslr. Members of these organizations have the opportunity to enroll in Counslr and access Counslr-provided services through the firm’s mobile platform. Members have no cost-sharing elements or usage limitations. Users’ information is private and confidential as required by the Health Insurance Portability and Accountability Act (HIPAA). The app and its data are encrypted.

## Methods

### Overview

Data used for this retrospective study included Counslr users from October 26, 2021 to November 22, 2023 and focused exclusively on Counslr’s text-based, real-time support sessions. This period covers the initial start-up of Counslr and its corresponding initial user membership. Counslr users, in this study, included students who accessed Counslr through their schools, universities, or colleges’ partnerships with Counslr and employees who accessed Counslr through their organizations’ partnerships with Counslr. Counslr provided a retrospective, deidentified dataset for this study. The provided data originated from user-reported information and data generated within the Counslr platform. Before a support session begins, users complete a survey where they self-report information to assist their counselor during the session. Second, data are generated within the platform such as the date or time of the session, session type (eg, on-demand or scheduled), and session length.

The session is the unit of analysis in this study. The data did not include a variable to identify individual users; thus, adjustments were not made for users who had multiple sessions. Individual datasets were provided for many of the variables. In some instances, dates were not consistent across the individual datasets (eg, a date missing in one dataset), and in other instances, presumable entry errors were present (eg, a session length was entered in minutes instead of a percentage of an hour). We adjusted for these challenges by either dropping the date from the dataset or adjusting the error where possible.

The primary purpose of this study was to evaluate how people access a 24-hour, digital mental health support app, where people communicate in real-time with human counselors through text messaging, and whether access patterns differ by session type (on-demand or scheduled) and membership type (education or noneducation). Access patterns were evaluated by day and hour (reported to authors in hour blocks [eg, 7 AM and 8 AM], not a specific time). The day and hour session distributions were stratified, separately, by session and membership types to examine whether access patterns differed between on-demand and scheduled sessions and between education- and non–education-based users. Chi-square tests were used for these comparisons. Chi-square tests with Bonferroni corrections were used as post hoc tests when appropriate.

The second evaluation used 2-tailed *t* tests to examine if session length (minutes) statistically differed between session types as well as by membership types. The last examination concerned users’ self-reported reasons for initiating their sessions. Users could select a reason or reasons for their sessions from a set list provided within the app or provide their own reason for initiating a session. The self-reported categories or reasons for initiating sessions provided in the data included academic concerns; anxiety; depression; emotional abuse; family issues; infidelity; lesbian, gay, bisexual, and transgender (LGBT); relationship reasons; substance use; no reason provided (none); and others. In addition, 3 other categories were reported that were combined with these more general categories: “depression or anxiety” (n=3) was placed in the depression category; “other or relationship or depression” (n=1) was placed in the other category; and “[relationship]” (n=3) and “relationship or depression” (n=1) were placed in the relationship category. Overall summary statistics were estimated for the session reasons. In addition, the session reasons were tabulated and stratified by session type and by membership type. Chi-square tests were used to evaluate whether statistical differences existed between session types and, separately, between membership types in terms of why users initiated their sessions.

### Ethical Considerations

The data used in this study were collected during Counslr’s standard operations and adhered to HIPAA guidelines. The data are not publicly available. Hofstra University’s institutional review board guidelines [19] deemed this study as non–human subject research because (1) Counslr provided a deidentified secondary dataset to the authors for evaluation, (2) the authors did not participate in the data collection and were not able to link the individuals in the deidentified dataset to living individuals through a code, and (3) the authors had independent control over how the data were analyzed and the study’s conclusions.

## Results

In total, 2 samples were created for the study due to the information available within each individual dataset. The primary dataset consisted of 327 support sessions that occurred between October 26, 2021 and October 19, 2023. The second sample consisted of the reasons why people initiated support sessions; this dataset consisted of support sessions conducted between August 15, 2023 to November 22, 2023 and, unlike session reasons provided for earlier dates, permitted stratification by both membership type and session type. For both datasets, dates without sessions were not included as were dates with data challenges that we could not address. The majority of evaluated sessions (206/327, 63%) were on-demand. Education-based users were the primary users in the sample (283/327, 86.5% of the sessions); the remaining sessions (44/327, 13.5%) comprised people associated with non–education-based memberships. There was no statistical difference (*P*=.93) in how education and noneducation members accessed Counslr; the majority of both groups (education: 178/283, 62.9%; noneducation: 28/44, 63.6%) accessed Counslr through on-demand sessions.

Across session and membership types, the mean and median session lengths were 40 (SD 15.3) and 45 minutes. Three sessions were noted as 0 minutes, while the longest session was 76 minutes. Mean session lengths for education- and non–education-based users were not significantly different (*P*=.57); the mean session lengths for education- and non–education-based users were 39.8 (SD 15.4; median 45, IQR 36-48) and 41.2 (SD 14.4; median 45, IQR 42.5-48.5) minutes. Differing from membership type, mean session length did statistically differ (*P*=.001) between on-demand (37.9, SD 16.6; median 45, IQR 27-47 minutes) and scheduled sessions (43.5, SD 11.9; median 46, IQR 42-49 minutes).

The share of sessions allocated to Fridays, Saturdays, and Sundays were each below 14.29% (Table 1); 14.29% is the expected share for each day if sessions were evenly distributed across all 7 days. Conversely, the share of sessions allocated to Mondays, Tuesdays, Wednesdays, and Thursdays each exceeded 14.29%. The distribution of sessions across days did not statistically differ (*P*=.62) by membership type. However, this distribution did statistically differ (*P*=.03) by session type. In post hoc tests, the distributions of session type on Thursdays and Sundays were seemingly different relative to the same distribution on other days, yet, after Bonferroni corrections, no statistical differences were present at the Bonferroni-corrected significance level of .007.

**Figure 1:**
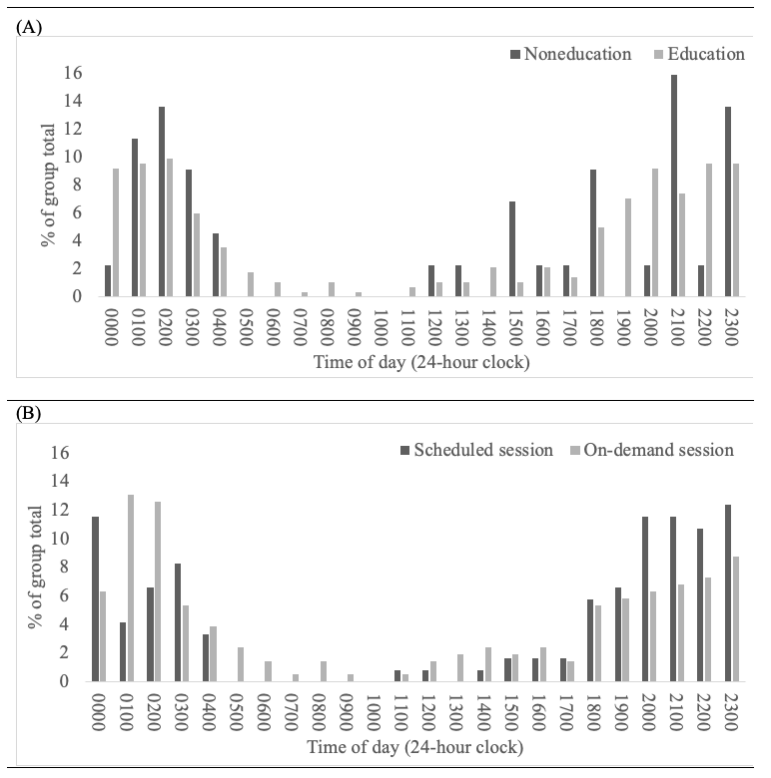
Distribution of user sessions by hour: hour of session by (A) membership type (noneducation or education) and (B) session type (scheduled or on demand). The data provided to the authors noted hours in blocks (eg, 7 AM, 8 AM) not specific times. Sessions from October 26, 2021 to October 19, 2023 (n=327); χ2 test for membership type group distribution difference: *P*=.19; χ2 test for session type group distribution difference: *P*=.10.

From a time perspective (Figure 1), 80.1% (262/327) of sessions were documented (in hour blocks, not specific times) as occurring from 7 PM to 5 AM, 41.6% (136/327) of sessions occurred between 7 PM and 12 AM, while 38.5% (126/327) of sessions were between 12 AM and 5 AM. This pattern was also present by membership type as 80.9% (229/283) of education-based users and 75% (33/44) of non–education-based users accessed the platform between 7 PM and 5 AM. The distribution of sessions across hours of the day did not statistically differ between session type (*P*=.10) or membership type (*P*=.19). Figure 1 illustrates these hourly-based distributions by membership type (A) and session type (B).

As indicated, a second dataset was constructed to evaluate why users initiated support sessions. Users could select multiple reasons for initiating a session; we evaluated 327 reasons in this study. While this is the same number of sessions included in the first dataset, this dataset covers a shorter time span than the first dataset and the reasons users provided for their sessions in this second dataset are for a smaller number of sessions than 327. The top 3 self-reported reasons for initiating a session (Table 2) pertained to relationships (75/327, 22.9%), depression (71/327, 21.7%), and anxiety (62/327, 19%). Among non–education-based users, the top 3 reasons for initiating a session (Table 2) concerned relationships (23/64, 35.9% of sessions), anxiety (10/64, 15.6%), and family issues (9/64, 14.1%). Among education-based users, the top 3 reasons for engaging in a session were depression (65/263, 24.7%), anxiety (52/263, 19.8%), and relationships (52/263, 19.8%).

**Table 1:** Distribution of user sessions by day of the week^a^.

Day	All, n (%)	Membership type, n (%)		Session type, n (%)		
		Education	Noneducation	On demand	Scheduled	*P* value^b^
Monday	49 (15)	43 (15.2)	6 (13.6)	28 (13.6)	21 (17.4)	.36
Tuesday	58 (17.7)	46 (16.3)	12 (27.3)	32 (15.5)	26 (21.5)	.17
Wednesday	60 (18.4)	51 (18)	9 (20.5)	38 (18.5)	22 (18.2)	.95
Thursday	52 (15.9)	47 (16.6)	5 (11.4)	26 (12.6)	26 (21.5)	.03^c^
Friday	44 (13.5)	40 (14.1)	4 (9.1)	32 (15.5)	12 (9.9)	.15
Saturday	27 (8.3)	23 (8.1)	4 (9.1)	21 (10.2)	6 (5)	.10
Sunday	37 (11.3)	33 (11.7)	4 (9.1)	29 (14.1)	8 (6.6)	.04^c^
*P* value^d^			.62		.03	

^a^Sessions from October 26, 2021, to October 19, 2023 (n=327).

^b^*P* values from post hoc *χ*^2^ tests; the Bonferroni-corrected significance level for these tests is .007.

^c^These *P* values are considered above the Bonferroni-corrected significance level of .007 and therefore do not support a statistically significant difference between the percentages.

^d^*P* values from *χ*^2^ evaluations on overall distributions of sessions over days.

**Table 2:** Users’ self-reported reasons for sessions, by session type and membership type^a^.

Session reason	All, n (%)	Membership type, n (%)				Session type, n (%)		
		Education	Noneducation	*P* value^b^	On demand		Scheduled	*P* value^b^
Academic concerns	20 (6.1)	14 (5.3)	6 (9.4)	.23	14 (6.7)		6 (5.1)	.58
Anxiety	62 (19)	52 (19.8)	10 (15.6)	.45	39 (18.6)		23 (19.7)	.81
Depression	71 (21.7)	65 (24.7)	6 (9.4)	.01	53 (25.2)		18 (15.4)	.04
Emotional abuse	4 (1.2)	2 (0.8)	2 (3.1)	.12	3 (1.4)		1 (0.9)	.65
Family issues	23 (7)	14 (5.3)	9 (14.1)	.01	13 (6.2)		10 (8.6)	.42
Infidelity	3 (0.9)	3 (1.1)	0 (0)	.39	3 (1.4)		0 (0)	.19
LGBT^c^	10 (3.1)	8 (3)	2 (3.1)	.97	7 (3.3)		3 (2.6)	.70
No reason provided (none)	11 (3.4)	11 (4.2)	0 (0)	.10	0 (0)		11 (9.4)	<.001
Other	42 (12.8)	37 (14.1)	5 (7.8)	.18	29 (13.8)		13 (11.1)	.48
Relationship	75 (22.9)	52 (19.8)	23 (35.9)	.01	48 (22.9)		27 (23.1)	.96
Substance use	6 (1.8)	5 (1.9)	1 (1.6)	.86	1 (0.5)		5 (4.3)	.01

^a^Reasons (n=327) provided during sessions from August 15, 2023 to November 22, 2023. Users could select a reason or reasons for the session from the list or type another reason or reasons.

^b^*P* values from *χ*^2^ tests.

^c^LGBT: lesbian, gay, bisexual, and transgender.

As illustrated in Table 2, a statistical difference was present in the percentage of education and noneducation users who accessed support sessions for depression (*P*=.01), family issues (*P*=.01), and relationships (*P*=.01). Table 2 also illustrates the reasons users initiated sessions by session type. The primary reasons for support sessions by session type were depression (53/210, 25.2%), relationships (48/210, 22.9%), and anxiety (39/210, 18.6%) for on-demand sessions and relationships (27/117, 23.1%), anxiety (23/117, 19.7%), and depression (18/117, 15.4%) for scheduled sessions. Users accessed support through scheduled sessions (5/117, 4.3%) at a statistically (*P*=.01) greater percentage relative to on-demand sessions (1/210, 0.5%) for substance use (as well as for the reason of none or no reason provided). Conversely, users accessed support for depression at a statistically (*P*=.04) higher percentage through on-demand sessions (53/210, 25.2%) relative to scheduled sessions (18/117, 15.4%).

## Discussion

### Principal Findings

Digital mental health apps have the potential to expand access to mental health services. These apps can help people overcome barriers to obtaining such care [6-8]. In this study, we analyzed Counslr, a digital mental health app that offers real-time, text-based support sessions with human counselors. We found that users who accessed Counslr participated in support sessions for an average of 40 minutes. The majority of these sessions occurred during times when standard, human-based mental health support is not available; this salient finding illustrates the promise of human-based, digital mental health apps in overcoming structural barriers to accessing mental health support services.

The novelty of this study in terms of evaluating user engagement with a human-based, nonscripted app, where users engage with human counselors through text messaging in a real-time, nonexperimental setting, prevents multiple, direct comparisons with the peer-reviewed literature. However, a few comparisons on similar metrics illustrate how usage patterns may differ between app designs. For instance, differing from this study’s result that the majority of sessions occurred between 7 PM and 5 AM, Booth et al [14], evaluating the usage of a chatbot, found that peak interaction hours with the chatbot were in the hours of 8-10 AM, 1 PM, and 5 PM. However, closer to this study’s results, Baumel et al [13]—evaluating 93 apps—found that usage peaked at the 8 PM hour for the apps included in their mental health category, while apps in their mindfulness and meditation category peaked in the hours of 7-9 AM and 10 PM to 12 AM. Baumel et al [13] also illustrated, in a graph, that usage rates for apps in their mental health category were highest on Tuesdays, Wednesdays, and Thursdays, which is similar to this study’s overall, nonstratified results (statistical comparisons were not conducted on this study’s overall, nonstratified day of the week statistics); the peak day of usage for the mindfulness or meditation applications Baumel et al [13] reviewed was Thursday.

Raue et al [17], evaluating a text-based app that included human interactions (users could text at any time in their study, while therapists reviewed the texts during working hours and responded at least 1 time a day to a person’s text 5 days a week; [17], page 3), did not report directly comparable metrics to those illustrated in this study. However, from a time of session perspective, Baumel et al [13] found that the median daily minutes with which active users engaged in 2 peer-supported apps they reviewed was 35.08; a finding below the 45-minute median found in this study. Overall, Baumel et al [13] found that the median daily minutes of use per active app user was different between mental health apps (10.02 minutes), happiness apps (7.77 minutes), and mindfulness and meditation apps (21.47 minutes), demonstrating that the reason why people engage with an app may influence usage.

The primary reasons users of Counslr self-reported for initiating sessions were similar to mental health conditions found across the population. For instance, 2 of the primary reasons Counslr users noted for initiating sessions, anxiety and depression, were also the most prevalent mental health conditions found among college students [20], while symptoms related to anxiety disorders or depressive disorders were present in 20.7% of US adults in early 2024 [21]. Collectively, how Counslr users engaged with licensed counselors outside of standard office hours, coupled with the commonality between mental health conditions found in the general population and the reasons Counslr users initiated support sessions, provide evidence that people will use a human-based, digital mental health app to access support outside of standard hours for prevalent mental health conditions.

While we did not have extensive demographic information about the Counslr users whose sessions were evaluated within this study, the 2 different membership types allowed comparisons between people who accessed the platform as students and those who accessed the platform as employees. When examined, usage patterns between these 2 groups did not statistically differ. Both students and employees engaged in sessions primarily between 7 PM and 5 AM and had similar patterns in terms of the days on which their sessions were held. Furthermore, the majority of both student and employee users accessed the platform using the on-demand feature instead of scheduling an appointment. The access patterns found among both student and employee users indicate that mental health support apps have the potential to increase access to mental health support services across multiple groups. This potential, universal appeal of Counslr-like platforms is also seen in how the platform is able to provide support for a wide array of reasons: from anxiety and depression to infidelity and academic concerns.

As noted here and elsewhere [9], the 24-hour accessibility of mental health apps is one attribute of such apps that could decrease structural barriers to accessing support services to address mental health challenges. However, the salient findings here regarding on-demand and scheduled sessions demonstrate that how access to such apps is provided is also important in terms of app design. For instance, there were no statistical differences between the frequency of on-demand and scheduled sessions in terms of how the sessions were distributed across the hours of the day; people both scheduled sessions and initiated on-demand sessions at 4 AM, indicating a need for both types of sessions.

Differences between the 2 session types also illustrate the need to consider offering both on-demand and scheduled sessions within a digital mental health support app; doing such may help facilitate the apps’ ability to improve access to mental health support. For instance, the distribution of sessions across days of the week differed between on-demand sessions and scheduled sessions, indicating possible user preferences for set sessions with counselors or on-demand sessions on certain days. Furthermore, a larger share of sessions initiated for the reason of substance use occurred as scheduled sessions relative to on-demand sessions. This finding suggests that users may prefer scheduled sessions for substance use relative to on-demand sessions, indicating that during the app design stage, developers should consider whether available access modes (on-demand and scheduled) address how users may prefer different access points depending on the specific reason they are accessing the app.

The connection between app design and this study’s results was possible because Counslr shared their formative data with researchers for independent analysis. As Counslr and other platforms continue to share their data for analyses, additional insights into future app development may arise; these insights may result in increased efficiency in app development as well as the creation of platforms designed to address the needs of users, thereby possibly increasing usage among people who seek mental health support thought such apps.

In this study, we conducted a novel evaluation of a 24-hour, real-time, digital mental health support app where users interact with humans through text messaging. The data were from a nonexperimental situation, helping to address the stated need for more information regarding how digital mental health apps perform outside of experimental settings [6,13,15,16]. While the findings of this study illustrate the promise of digital mental health support apps to increase access to a variety of mental health needs, a number of limitations are present. First, this study relied on a small, formative dataset provided by Counslr for independent analysis. While a larger sample size is preferable, the study allowed multiple insights into usage patterns and why people accessed the platform. Furthermore, using this formative dataset to evaluate initial platform usage allowed the authors to suggest additional metrics for data collection and analysis to the organization.

A second limitation is that the deidentified data used here and the lack of demographic variables entail that the findings of this study only pertain to study participants and are not generalizable to the overall population. Furthermore, the data did not include individual identifiers, preventing adjustments for users with multiple sessions, which could skew the results (eg, if one user initiated multiple sessions for the same reason). To provide further insight into this limitation, Counslr, at the authors’ request, provided information illustrating that there were 154 unique users in the data with an average and median number of sessions of 2.06 and 1 (minimum 1 and maximum 21).

The deidentified nature of the data and the manner in which the data were provided to the authors also prevented more complex data analysis (eg, regression analysis), which could provide further insights into usage and access patterns. In addition, the platform did not collect demographic information on users such as how they identify their ethnicity, gender, and race. The inclusion of such information would allow pivotal evaluations regarding whether digital mental health support apps can help address disparities in access to mental health support services across different demographic groups. Finally, while the primary purpose of this study was to evaluate how and why people engage with licensed counselors through a digital, real-time, text-based mental health support app in nonexperimental settings, future evaluations—when such data are available—must examine the effectiveness of such apps in addressing the mental health support needs of users and whether such platforms serve as a complement to more traditional mental health services.

### Conclusion

Barriers that prevent people from accessing mental health support services are multiple and include structural barriers and stigma. Anonymity and 24-hour access to digital mental health support apps may help address such barriers. In this study, we evaluated how people use Counslr, a real-time mental health support app where users engage with licensed, human counselors through text messaging sessions. The majority of both education- and non–education-based users initiated mental health support sessions outside of standard business hours for a variety of reasons, indicating the promise of Counslr like apps to address barriers to accessing mental health support services. The similarities in usage patterns of Counslr student and employee users, despite one group being noneducation based and the other being education based, demonstrated an almost universality in the platform in terms of serving multiple groups with multiple needs.

The access patterns found in this study demonstrate the promise of Counslr-type apps to help address structural barriers to accessing mental health support services. Additional research is needed to evaluate the effectiveness of such apps, notably human-based apps such as Counslr, and whether such apps can also address disparities in access to mental health support services among demographic groups.

## Abbreviations

HIPAA: Health Insurance Portability and Accountability Act
LGBT: lesbian, gay, bisexual, and transgender

## Data Availability

The data analyzed in this study are not publicly available. The data were provided to the authors by Counslr.
